# Recent advances in understanding of chronic kidney disease

**DOI:** 10.12688/f1000research.6970.1

**Published:** 2015-11-04

**Authors:** Junna Yamaguchi, Tetsuhiro Tanaka, Masaomi Nangaku

**Affiliations:** 1 Division of Nephrology and Endocrinology, The University of Tokyo Graduate School of Medicine, Tokyo, 113-0033, Japan

**Keywords:** Chronic Kidney Disease, Hypoxia, Fibrosis, Tubular Atrophy, Pathogenesis, Nephrogenesis

## Abstract

Chronic kidney disease (CKD) is defined as any condition that causes reduced kidney function over a period of time. Fibrosis, tubular atrophy and interstitial inflammation are the hallmark of pathological features in CKD. Regardless of initial insult, CKD has some common pathways leading CKD to end-stage kidney disease, including hypoxia in the tubulointerstitium and proteinuria. Recent advances in genome editing technologies and stem cell research give great insights to understand the pathogenesis of CKD, including identifications of the origins of renal myofibroblasts and tubular epithelial cells upon injury. Environmental factors such as hypoxia, oxidative stress, and epigenetic factors in relation to CKD are also discussed.

## Introduction

Chronic kidney disease (CKD) is a growing health burden with an increasing incidence and prevalence worldwide. An estimated 13% of adults in the US and Japan have CKD, and the proportion of affected individuals increases each year because of an aging population and increases in diabetes and hypertension, the most common causes of CKD ^1, 2^. CKD is a risk factor for end-stage kidney disease (ESKD), cardiovascular disease, and overall mortality ^3^. In the US, the economic costs of CKD and ESKD in patients over age 65 are $60 billion, representing 24% of total Medicare expenditures in 2011 ^4^. Currently, the predominant problem is that therapeutic options for CKD are limited and often ineffective, meaning that there is essentially no cure for CKD. Therefore, translating our understanding of CKD pathogenesis into treatments is a high priority in the field.

CKD is defined as any condition that causes abnormalities of kidney structure or function for a duration of more than 3 months with notable implications for patient health ^5, 6^ ( Table 1). Regardless of initial etiology, fibrosis, tubular atrophy, and interstitial inflammation are common pathological features of CKD. Careful histological observations have demonstrated that functional impairment of the kidney is more highly correlated with tubulointerstitial damage than with glomerular injury, which is often associated with the loss of peritubular capillaries (PTCs) ^7^. In addition, hypoxia is now accepted to be the final common mechanism underlying the progression of CKD to ESKD, which we discuss later in this article ^8, 9^.

**Table 1.** Definition of chronic kidney disease (KDIGO 2012).

Criteria for chronic kidney disease ^a^	Definition of criteria
One or more marker of kidney damage	Albuminuria (AER of ≧30 mg per 24 hours and ACR of ≧30 mg/g) Urine sediment abnormalities Electrolyte and other abnormalities due to tubular disorders Abnormalities detected by histology Structural abnormalities detected by imaging History of kidney transplantation
Decreased GFR	GFR of less than 60 ml/min per 1.73 m ^2^

^a^Either of the criteria below should be present for more than 3 months. Data are from the KDIGO (Kidney Disease: Improving Global Outcomes) 2012 Clinical Practice Guideline for the Evaluation and Management of Chronic Kidney Disease. ACR, albumin-to-creatinine ratio; AER, albumin excretion rate; GFR, glomerular filtration rate.

The current understanding of CKD is based on a broad range of studies focused on the genetic risk factors for the development and progression of CKD, the pathogenesis of renal fibrosis (e.g., the origin and activation of renal myofibroblasts, fibrogenic mediators and signaling, crosstalk with tubular cells, vasculature, and inflammatory cells), tubular injury and repair, mediators and dynamics of renal inflammation, and cellular adaptations to the microenvironment such as hypoxia and oxidative stress. This article reviews some of the recent advances in our understanding of CKD from two vantages: cellular regeneration and hypoxia. A better understanding of CKD pathogenesis will hopefully provide insights leading to better management of CKD in the future.

## Kidney development and regeneration

### Nephrogenesis and nephron number

Nephrogenesis requires precise sequential and reciprocal interactions between renal progenitor cells and their integration with vasculature. In mammals, the metanephric kidney develops through interactions between the metanephric mesenchyme (MM) and uretic bud ^10^. MM nephron progenitors give rise to Six2 ^+^ cap mesenchyme progenitor cells (which later differentiate into nephron epithelia, including proximal and distal tubular cells, the loop of Henle, and podocytes) and Foxd1 ^+^ cortical stromal progenitor cells (which later differentiate into cortical and medullary interstitial cells, mesangial cells, and pericytes) ^11– 14^. Nephrogenesis ceases at approximately the third post-natal day in mice ^15^ and 36 weeks of gestation in humans ^16^. Low nephron number is associated with a risk of renal disease and hypertension ^17^, and low birth weight and prematurity are the most robust clinical surrogates for low nephron number ^18^. The molecular event that governs the end of nephron formation is unknown and is an ongoing topic of research ^10, 19^. The regenerative capacity of glomeruli is limited after birth, and many studies have focused on the source of regenerated tubular cells following acute kidney injury (AKI) and the origin of myofibroblasts in CKD.

### Origin and regeneration of tubular cells

A proliferative burst of tubular cells occurs during kidney injury. Sophisticated lineage-tracing studies have excluded the possibility of extrarenal cells contributing to tubular regeneration ^20^. Recent studies further support the self-proliferation of existing differentiated tubular cells rather than the contribution of stem-like cells to epithelial proliferation after AKI ^21– 23^.

Whereas the origin of the repairing tubule is becoming clearer, less is known regarding the signals that regulate epithelial dedifferentiation, proliferation, and polarization. One signal is known to derive from inflammatory cells. Cellular stress in tubules induces the activation of innate immunity through the production of cytokines and chemokines, which exacerbate tubular injury by recruiting macrophages, neutrophils, and proinflammatory lymphocytes ^24^. One study demonstrates that a lack of interleukin-1 receptor-associated kinase-M leads to persistent proinflammatory macrophage infiltration with higher tubular phagocytosis activity and thus limited tubular re-epithelialization ^25^. This effect was reversed by tumor necrosis factor-alpha blockade, indicating that cytokine-induced tubular attack overwhelms tubular repair capacity. Advances in genetic manipulation at the desired time point, together with a better understanding of myofibroblasts, now allow the study of signaling from fibroblasts or myofibroblasts to tubular cells *in vivo*.

However, whether tubular regenerative capacity is itself limited in CKD is unknown. The intrinsic limit of tubular regenerative capacity may be related to disturbances in metabolism, endoplasmic reticulum stress, cell cycle arrest, or DNA damage ^26^. In addition, the first direct reprogramming of renal epithelial cells to Six2 ^+^ nephron progenitor cells was accomplished by the addition of a combination of six transcription factors, including SIX2 and OSR1 ^27^. These reprogrammed cells differentiated into epithelial cells in a re-aggregation assay, providing another strategy for replacing the epithelial layer if correct integration into nephrons can be achieved. In parallel, several groups have succeeded in the induction of cells of renal lineage, including intermediate mesoderm as well as individual differentiated cells such as proximal tubular cells or podocytes from embryonic stem or induced pluripotent stem cells ^11, 28, 29^. Similar to the maintenance of nephron progenitor potency in the stromal-epithelial niche during kidney development, sophisticated programs may be required to maintain this potency ^30^.

### Origin of myofibroblasts and their transdifferentiation

Myofibroblasts are extracellular matrix-producing cells that drive fibrogenesis. The origin of renal myofibroblasts has been another area of major debate. Currently, FoxD1-Cre-labelled pericytes ^31^, P0 (myelin protein 0)-Cre-labelled resident fibroblasts ^32^, and renal erythropoietin-producing (REP) cells ^33^ are reported as the origins of myofibroblasts. The absence of permanent specific markers and a shared developmental program makes it difficult to determine their precise origin. Their similar localization—near CD31 ^+^ endothelial cells in the interstitium—and gene expression patterns (PDGFRβ (platelet derived growth factor receptor beta) and CD73) suggest that they represent an overlapping cell population. A recent study reported that Gli1 ^+^PDGFRβ ^+^CD73 ^−^ cells, a small fraction of the total PDGFRβ population, are the major cellular origin of myofibroblasts in multiple organs, including kidney, heart, and liver ^34^. Unified theories require further investigation.

Triggers of the transdifferentiation of resident fibroblasts, REPs, or pericytes to alpha-smooth muscle actin-producing myofibroblasts also remain unclear. Factors produced by injured tubular and inflammatory cells, including vascular endothelial growth factors (VEGFs), platelet-derived growth factors (PDGFs), fibroblast growth factors, and transforming growth factor-beta, activate pericytes and induce their detachment from capillaries and their transdifferentiation to myofibroblasts ^33, 35^. In a typical inflammatory fibrogenic model known as unilateral ureteric obstruction (UUO), this transdifferentiation was found to be partially reversible in REPs after removal of the insult ^33^. Recently, a comprehensive DNA microarray analysis of pericyte-to-myofibroblast transition was performed by using translational ribosome affinity purification in UUO, which may yield clues to help characterize these cells ^36^.

## Mediators of chronic kidney disease progression

### Proteinuria

Proteinuria is an established mediator of CKD pathogenesis, and lowering proteinuria retards CKD progression ^37– 40^. Protein overload exacerbates tubulointerstitial injury in a number of ways: direct tubular injury, including lysosomal rupture and energy depletion; activation of intratubular complement components, which leads to tubular cell activation or injury; and stimulation of inflammatory and fibrogenic mediators ^41– 43^.

### Hypoxia

**Figure 1.**
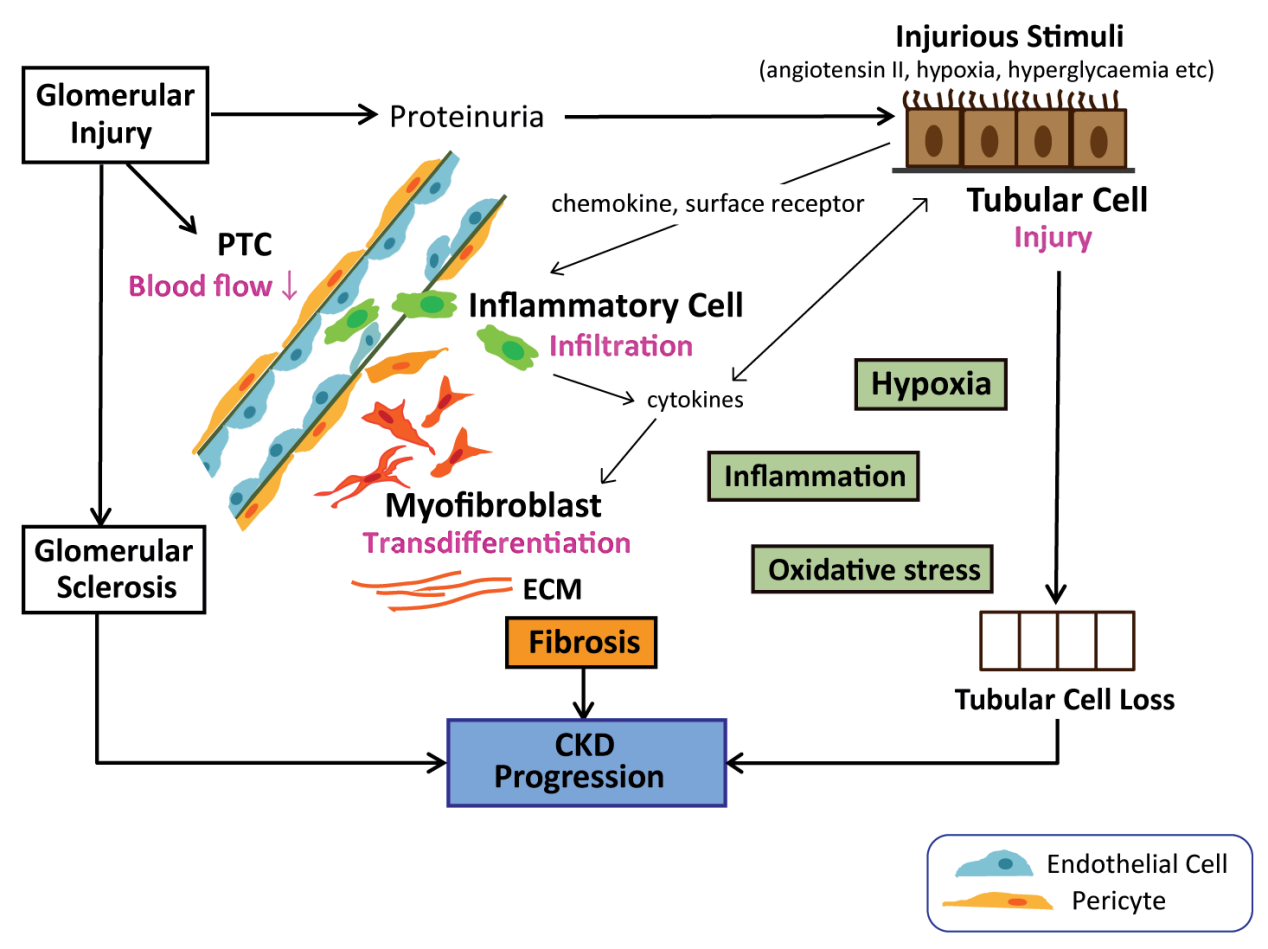
Pathogenesis of chronic kidney disease. Tubulointerstitial hypoxia, inflammation, and oxidative stress form a vicious cycle in chronic kidney disease (CKD) progression. Glomerular injury results in a decrease in peritubular capillary (PTC) blood flow and subsequent tubulointerstitial hypoxia. Hypoxia and proteinuria cause tubular injury, which in turn triggers the production of cytokines and chemokines and promotes inflammatory cell infiltration into the tubulointerstitium. Damaged PTC also facilitates inflammatory cell infiltration. Hypoxia, inflammation, and oxidative stress promote the transdifferentiation of resident fibroblasts, renal erythropoietin-producing cells, or pericytes to extracellular matrix (ECM)-producing myofibroblasts. Direct interactions between the injured tubular cells and myofibroblasts also play a role. Fibrosis further impairs local oxygenation.

The fact that nonproteinuric CKD is common and that renin-angiotensin-aldosterone inhibitors have renoprotective effects beyond lowering blood pressure and reducing proteinuria suggests that there are other key mediators of CKD pathogenesis. Chronic hypoxia of the tubulointerstitium is now widely accepted as the final common pathway in CKD progression ^8, 9^ ( Figure 1). Once PTC rarefaction occurs, hypoxia in the affected region triggers phenotypic changes in tubular cells (e.g., proliferation rate and apoptosis), which in turn serve as a source of mediators involved in inflammatory cell infiltration and fibrosis. Fibrosis further impairs local oxygenation, while hypoxia induces sterile inflammation. Hypoxic responses are also induced by inflammatory transcription factors ^44^. Thus, hypoxia is intricately linked to inflammation and oxidative stress, causing a vicious cycle leading to CKD pathogenesis.

Hypoxia-inducible factors (HIFs) are transcription factors that function as master regulators of biological adaptive responses to hypoxia ^45^. HIFs consist of an alpha subunit (HIF-1α, HIF-2α, and HIF-3α) and a common beta subunit. Under normoxic conditions, HIF-α is hydroxylated by prolyl hydroxylase (PHD) and undergoes proteasomal degradation. HIFs regulate the expression of more than 150 genes, including those involved in anaerobic metabolism (e.g., glucose transporter-1), hematopoiesis (erythropoietin, or EPO), and angiogenesis (e.g., VEGF and angiopoietins). In response to hypoxia in kidney, HIF-1α is expressed in tubular cells, whereas HIF-2α is expressed mainly in endothelial cells and interstitial fibroblasts ^46^.

In kidney disease, despite the hypoxic milieu, HIF activation is considered to be suboptimal. In the early phase of UUO (day 2), induction of HIF-1α and its target genes was disrupted, although pronounced hypoxia was confirmed by a hypoxia-detecting probe ^33^. In another study using a rat CKD model, indoxyl sulphate, a representative uremic toxin, impeded the recruitment of transcriptional coactivators to HIF-1α, causing insufficient upregulation of HIF-1 target genes while leaving HIF-1α protein level unaffected ^47^. This was reversed by an oral adsorbent for CKD, AST-120, that is currently in clinical use. Indeed, genetic and pharmacological modulation of HIFs in the kidney has been a subject of great interest, not only for investigating the roles of HIFs but also as a potential therapeutic tool. The renoprotective effects of HIF activation have been demonstrated in various AKI models, whereas those in CKD models have variable outcomes ^48^. Pepck-Cre-mediated conditional knockout of HIF-1α in proximal tubules ameliorated fibrosis in UUO ^49^, whereas global HIF activation by *Vhl* knockout ameliorated inflammation and fibrosis in the same model ^50^. Global HIF activation by PHD inhibition reduced the tubulointerstitial injury associated with reduced tubular injury and capillary rarefaction in CKD rats ^51^ and improved oxygen metabolism in diabetic rats ^52^. HIF-1 in tubular cells exhibits both autocrine (e.g., cell cycle regulation and metabolic regulation) and paracrine (e.g., angiogenic and fibrogenic factors) signaling, which may result in different long-term renal outcomes. Additional cell type-specific and time-dependent manipulations of HIF activity may yield further insight for the development of future kidney therapies.

Renal anemia is a frequent complication of CKD. The pathogenesis of renal anemia includes chronic inflammation, iron deficiency, shortened erythrocyte half-life, and, most importantly, EPO deficiency. One explanation for the observed EPO deficiency is the accumulated indoxyl sulphate observed in CKD. Indoxyl sulphate is reported to suppress EPO production in a HIF-dependent manner ^53^. The identification of REPs also provided insight into the causes of EPO deficiency. REPs were repressed of EPO producing potential upon transdifferentiation to myofibroblasts in UUO through the activation of nuclear factor-kappa-B (NF-κB) signals ^33^. REP-specific PHD2 knockout mice recovered EPO production in UUO and lipopolysaccharide-treated mice via HIF-2 activation ^54^. This finding is in accordance with the observation that the pharmacological activation of HIFs by PHD inhibitors augmented EPO production in patients with ESKD ^55^. Notably, PHD2 knockout-mediated HIF activation in REPs did not affect the inflammatory or fibrotic pathology of UUO; REP plasticity seems to be regulated by multiple signals at multiple levels.

What causes angiogenesis insufficiency in CKD? Hypoxia signals generally promote angiogenesis ^56^, and PTC development is thought to be regulated by angiogenic factors (e.g., VEGF, fibroblast growth factors, angiopoietins, and PDGF) secreted from tubular cells as well as endothelial and mesenchymal precursors. Doxycycline-regulated tubular-specific VEGF-A deletion during development led to a marked reduction of PTC, whereas deletion of VEGF-A post-natally between days 21 and 42 did not result in pronounced PTC rarefaction ^57^. This suggests a difference in tubulovascular crosstalk in the developing and adult kidney. Another study that focused on pericyte-endothelial crosstalk in the adult kidney ^58^ showed that PDGFβ and VEGF receptor signaling induced pericyte detachment from PTC and their transdifferentiation to myofibroblasts in UUO. These unusual behaviors by angiogenic factors may in part explain the insufficient angiogenesis in adult kidneys, including CKD kidneys.

### Oxidative stress

Oxidative stress, another type of oxygen disturbance, is inevitably present in CKD and inseparably linked to hypoxia and inflammation ^59^ ( Figure 1). Oxidative stress is caused by increased reactive oxygen species (ROS) production or impaired antioxidant capacity, or both. Factors such as proteinuria, uremic toxin, hyperglycemia, and increased activity in the intra-renal angiotensin system contribute to increased oxidative stress in CKD. The Keap1-Nrf2 (Kelch-like ECH-associated protein 1-nuclear factor-erythroid-2-related factor 2) system is the major regulator of cytoprotective responses to endogenous and exogenous stresses caused by ROS. Impaired Nrf2 activity is observed in various animal CKD models, and the activation of Nrf2 ameliorates antioxidant defense and inflammation. Pharmacological activation of the Nrf2 pathway has been challenged with synthetic triterpenoid bardoxolone methyl in type 2 diabetic CKD patients. A phase 2 BEAM (52-Week Bardoxolone Methyl Treatment: Renal Function in CKD/Type 2 Diabetes) trial showed promise for the use of bardoxolone methyl to increase estimated glomerular filtration rate (eGFR) compared with a placebo (mean change of 8.2 to 11.4 ml/min per 1.73 m ^2^, depending on the dose group) in moderate-to-severe diabetic CKD patients ^60^ (eGFR 20 to 45 ml/min per 1.73 m ^2^). Notably, increased albuminuria was observed in the bardoxolone methyl group, despite significantly improved kidney function. A study in cynomolgus monkeys suggests that bardoxolone methyl decreases the expression of megalin, which is primarily responsible for albumin reabsorption in proximal tubules, resulting in increased albuminuria ^61^. Whether and how Nrf2 is related to reduced megalin expression remain unknown. The subsequent phase 3 BEACON (Bardoxolone Methyl Evaluation in Patients with Chronic Kidney Disease and Type 2 Diabetes Mellitus: the Occurrence of Renal Events) trial in diabetic CKD stage 4 patients (eGFR of 15 to less than 30 ml/min per 1.73 m ^2^) was terminated because of a higher rate of cardiovascular events in the bardoxolone methyl group than in the placebo group ^62^. Controversies exist as to the cause of increased cardiovascular events during bardoxolone methyl treatment and as to the appropriate selection of a target patient population for this therapy ^63, 64^. Interventions designed to prevent oxidative stress remain important therapeutic options for CKD.

## New technology-driven advances in understanding of chronic kidney disease

### -Omics

‘-Omics’ approaches have rapidly expanded our understanding of CKD. Genome-wide association studies have identified multiple genetic loci associated with kidney function-related traits ^65– 68^. The shared loci among multiple ethnic groups include the *UMOD* locus, which encodes the abundant urinary protein uromodulin produced by the epithelial cells of the thick ascending limb of the loop of Henle (TAL). Further animal studies have demonstrated the causal role of *UMOD* risk variants in hypertension and CKD by modulating salt handling in the TAL ^69^. An example for a specific ethnic group is *APOL1*. The higher incidence of ESKD in African Americans compared with European Americans led to the identification of *APOL1* variants as risk factors for the development and progression of CKD among African Americans in the general population ^70– 72^.

### Epigenetics

Epigenetic regulation in CKD is emerging as an important topic. As proposed in the “metabolic memory” theory of diabetic nephropathy, hypoxia may be remembered via epigenetic changes to play a crucial role in the pathogenesis of CKD. Epigenetic modifications include cytosine DNA methylation, noncoding RNA, and histone post-translational modification ^73^. Differentially methylated regions were observed in the cortical tubules of CKD patients and controls, especially in enhancer regions of key fibrotic genes ^74^. Microarray approaches have identified a number of potential microRNAs responsive in CKD animal models ^75^. MicroRNA-21 was shown to promote fibrosis by repressing peroxisome proliferator-activated receptor-alpha, by either germline deletion of miR21 or oligonucleotide administration of anti-miR21 in UUO ^76^. Hypoxia is also reported to alter the chromatin conformational structure dynamically and cause histone modifications in human umbilical vein endothelial cells, which result in transcriptional changes of HIF-1 target genes ^77^. Prolonged ischemic-reperfusion injury has caused histone modifications at proinflammatory and profibrotic genes prior to fibrosis, which may be related to CKD pathogenesis ^78^. Interventional studies for these epigenetic modifications are anticipated.

## Perspectives

Technological developments in genome editing, genome-wide analysis, and dynamic multiplex four-dimensional measurement, as well as advances in the fields of stem cell and regenerative biology are considerable. It is now possible to investigate the contextual, environmental, and interdependent coordination between multiple players in the kidney ^79^. Needless to say, translating the results of basic research in animal models to the bedside will require a number of additional studies. One example is the lack of animal models that mimic human CKD pathophysiology. To overcome these issues, research using samples from patients with CKD is under way. Overall, this is an exciting time for CKD research, as a fuller understanding of its pathogenesis lays the foundation for pathogenesis-based kidney therapy.

## Abbreviations

AKI, acute kidney injury; CKD, chronic kidney disease; eGFR, estimated glomerular filtration rate; EPO, erythropoietin; ESKD, end-stage kidney disease; HIF, hypoxia-inducible factor; MM, metanephric mesenchyme; Nrf2, Nuclear factor-erythroid-2-related factor 2; PDGF, platelet-derived growth factor; PDGFRβ, platelet-derived growth factor receptor beta; PHD, prolyl hydroxylase; PTC, peritubular capillary; REP, renal erythropoietin-producing; ROS, reactive oxygen species; TAL, thick ascending limb of the loop of Henle; UUO, unilateral ureteric obstruction; VEGF, vascular endothelial growth factor.
